# Current status and molecular epidemiology of rabies virus from different hosts and regions in Malawi

**DOI:** 10.1007/s00705-022-05635-z

**Published:** 2023-01-12

**Authors:** Henson Kainga, Elisha Chatanga, Marvin Collen Phonera, John Pilate Kothowa, Precious Dzimbiri, Gladson Kamwendo, Malala Mulavu, Cynthia Sipho Khumalo, Katendi Changula, Herman Chambaro, Hayato Harima, Masahiro Kajihara, Kholiwe Mkandawire, Patrick Chikungwa, Julius Chulu, Gilson Njunga, Simbarashe Chitanga, Benjamin Mubemba, Michihito Sasaki, Yasuko Orba, Yongjin Qiu, Junya Yamagishi, Edgar Simulundu, Ayato Takada, Boniface Namangala, Hirofumi Sawa, Walter Muleya

**Affiliations:** 1 Department of Veterinary Epidemiology and Public Health, Faculty of Veterinary Medicine, Lilongwe University of Agriculture and Natural Resources, PO Box 219, Lilongwe, Malawi; 2 Department of Disease Control, School of Veterinary Medicine, University of Zambia, PO Box 32379, Lusaka, Zambia; 3 Department of Veterinary Pathobiology, Faculty of Veterinary Medicine, Lilongwe University of Agriculture and Natural Resources, PO Box 219, Lilongwe, Malawi; 4 Laboratory of Parasitology, Graduate School of Infectious Diseases, Faculty of Veterinary Medicine, Hokkaido University, Hokkaido, 060-0818 Japan; 5 Department of Animal Health and Livestock Development, Ministry of Agriculture, PO Box 2096, Lilongwe, Malawi; 6 Department of Biomedical Sciences, School of Health Sciences, University of Zambia, PO Box 32379, Lusaka, Zambia; 7 Department of Biomedical Sciences, School of Veterinary Medicine, University of Zambia, PO Box 32379, Lusaka, Zambia; 8 Department of Paraclinical Studies, School of Veterinary Medicine, University of Zambia, PO Box 32379, Lusaka, Zambia; 9 Laboratory of Virology, Central Veterinary Research Institute (CVRI), Ministry of Livestock and Fisheries, 10101 Lusaka, Zambia; 10 Division of Molecular Pathobiology, International Institute for Zoonosis Control, Hokkaido University, Sapporo, 001-0020 Japan; 11 Division of Global Epidemiology, International Institute for Zoonosis Control, Hokkaido University, Sapporo, 001-0020 Japan; 12 Department of Veterinary Clinical Studies, Faculty of Veterinary Medicine, Lilongwe University of Agriculture and Natural Resources, PO Box 219, Lilongwe, Malawi; 13 Department of Paraclinical Studies, School of Veterinary Medicine, University of Namibia, Private Bag 13301, Windhoek, Namibia; 14 School of Life Sciences, College of Agriculture, Engineering and Sciences, University of KwaZulu-Natal, Durban, 4000 South Africa; 15 Department of Wildlife Sciences, School of Natural Resources, Copperbelt University, 50100 Kitwe, Zambia; 16 Department of Biomedical Sciences, School of Medicine, Copperbelt University, 50100 Ndola, Zambia; 17 Division of International Research Promotion, International Institute for Zoonosis Control, Hokkaido University, Sapporo, 001-0020 Japan; 18 Division of Collaboration and Education, International Institute for Zoonosis Control, Hokkaido University, Sapporo, 001-0020 Japan; 19 Macha Research Trust, 20100 Choma, Zambia; 20 Global Virus Network, Baltimore, ML 21201 USA; 21 One Health Research Center, Hokkaido University, Sapporo, 001-0020 Japan

## Abstract

Although rabies is endemic in Malawi, there have been no studies in which rabies virus was systematically investigated and characterized in multiple animal hosts in that country. In order to provide molecular epidemiological data on rabies virus in Malawi, 683 suspected rabies case reports from 2008 to 2021 were examined, and 46 (dog = 40, cow = 5, and cat = 1) viable rabies-positive brain samples archived at the Central Veterinary Laboratory (CVL), Lilongwe, Malawi, were analyzed genetically. The results showed an increase in the submission of brain samples from 2008 to 2010, with the highest number of submissions observed in 2020. Of the 683 case reports analyzed for the period under review, 38.1% (260/683) (CI: 34.44 – 41.84) were confirmed by direct fluorescent antibody test. Among the confirmed cases, 65.4% (170/260) (CI: 59.23 – 71.09) were canine rabies. Further, phylogenetic analysis revealed that sequences from different animal hosts clustered together within the Africa 1b lineage, suggesting that the strains circulating in livestock are similar to those in domestic dogs. This finding supports the hypothesis that canine rabies is spilling over to livestock and emphasizes the need for further studies to provide data for effective control of rabies in Malawi.

**Supplementary Information:**

The online version contains supplementary material available at 10.1007/s00705-022-05635-z.

## Introduction

Rabies is a devastating but neglected zoonotic disease caused by a negative-sense RNA virus of the genus *Lyssavirus*, family *Rhabdoviridae* [1]. Rabies causes acute viral encephalomyelitis that is invariably fatal in humans and other warm-blooded vertebrates such as dogs, cats, cattle, foxes, and jackals [2]. Although a wide range of animals can become infected and transmit the disease, only mammals of the orders Carnivora (jackals) and Chiroptera (bats) act as reservoirs for rabies virus (RABV) [3]. Rabies has a worldwide distribution, and in developing countries of Africa and Asia, human rabies incidence accounts for over 95% of global cases [4]. Globally, rabies claims 59,000 human lives annually, with an average of 21,476 fatal cases occurring in Africa [5, 6], with Tanzania reporting over 1,500 deaths annually [6, 7]. Approximately 40% of all human rabies deaths occur in children under the age of 15 years [2, 8]. However, these reported numbers might be too low due to underreporting.

The RABV genome encodes five structural proteins, namely nucleoprotein (N), phosphoprotein (P), RNA polymerase (L), matrix protein (M), and glycoprotein (G) [9–11]. The N gene is highly conserved and required for genome encapsidation, transcription, and replication [12]. It is therefore frequently used as a molecular marker in studies identifying the lineages of RABV that are circulating regionally and globally [13–17]. Within Africa, previously identified lyssaviruses include classical RABV, Lagos bat lyssavirus, Mokola lyssavirus, Duvenhage lyssavirus [18, 19], and, more recently, Ikoma and Shimoni lyssaviruses isolated in Kenya and Tanzania, respectively, in 2009 and Matlo bat lyssavirus isolated in South Africa [20, 21]. Classical RABV is the most common and comprises the Africa 1a, 1b, 2, 3, and 4 lineages [22–24], which are present in northern Africa, eastern and southern Africa, western and central Africa, southern Africa, and Egypt, respectively [16, 17, 19, 24–27]. In addition to the N gene, the P [28] and G [29] genes have also been used for genetic characterization. For studying genetic diversity, the G gene encoding the surface glycoprotein, which is responsible for viral attachment to host cells [30, 31] and thus a major target for host neutralizing antibodies, is preferred, as it is less conserved [31]. Its characteristics make the G gene a suitable target for phylogenetic comparison of closely related viruses. In regions separated by physical barriers, analysis of the G gene has demonstrated the separation of distinct viruses according to their respective geographical areas [32–34].

Malawi is a landlocked country in southeastern Africa covering an area of 118,484 km^2^. It is bordered by Mozambique to the southeast, Tanzania to the north, and Zambia to the west. Malawi is divided into three geographical regions (North, Central, and South) [35] consisting of 28 administrative districts and has an estimated human population of 19.13 million as of 2020 [36]. In Malawi, rabies is believed to be linked to domestic dogs (*Canis familiaris*), which transmit it to other species such as cattle (*Bos indicus*) and humans (*Homo sapiens*) through bites [37]. Thus, the human population faces a growing risk of RABV infection from domestic dogs, and several children have died due to rabies transmitted by dogs [38–41]. An increase in the human population and rural activities has led to increased encroachment into protected wildlife reserves. This has further increased the exposure of humans and domestic animals to wildlife and its pathogens, including RABV. Thus, the uncontrolled interaction between wildlife reservoirs and domestic dogs, coupled with a paucity of information on human rabies transmitted by carriers other than domesticated dogs, has further complicated the implementation of rabies control measures [39–41]. Although unconfirmed, domestic dogs are suspected to be a reservoir of RABV in Malawi [37, 41]. In other countries such as Mozambique, side-striped jackals (*Canis adustus*) and wild dogs (*Lycaon pictus*) are suspected reservoirs [42]. In Tanzania and Zambia, domestic dogs, black-striped jackals (*Canis mesomelas*), and side-striped jackals (*Canis adustus*) are considered maintenance hosts [17, 43].

By 2015, the burden of human rabies in Malawi was estimated to be 500 deaths per year, while economic losses estimated at 13 million United States dollars (USD) per year were attributed to investigations, diagnostics, control, vaccines, and livestock losses [5]. In Malawi, rabies diagnosis involves the use of a direct fluorescent antibody test (dFA) using fluorescein isothiocyanate (FITC)-labeled anti-RABV monoclonal immunoglobulin.

Despite mass rabies vaccination in Malawi, there are continual reports of human rabies cases associated with dog bites [37, 40, 44]. In order to alleviate the effects of rabies and implement effective control programs, there is a need to assess the current status of rabies and investigate its molecular epidemiology in different hosts and regions in Malawi. The lack of such information has negatively impacted rabies control strategies in the country. Thus, the aim of this study was to provide molecular epidemiological data on RABV by highlighting cases of rabies reported in domestic animals and wildlife and the extent of its diagnosis in Malawi. We also investigated the lineages and diversity of RABV in Malawi through analysis of the N and G genes, respectively. This information is vital for elimination of rabies in animals, which is a key step towards the ultimate reduction of the disease burden in humans and achieving the goal of having zero dog-mediated human rabies cases by 2030.

## Materials and methods

### Ethical considerations

Authorization and ethical clearance for the study (Ref: DAHLD/AHC/11/2021/1) were granted by the Animal Health Committee (AHC) of the Department of Animal Health and Livestock Development (DAHLD) in Malawi.

### Archived data collection

Rabies vaccination records for pets in Malawi were obtained from the epidemiological unit of the DAHLD. Case and diagnostic reports of rabies from 2008 to 2021, archived at the Central Veterinary Laboratory (CVL), Lilongwe, Malawi, were also collected. Confirmed cases were defined as those that were reported and from which brain tissue was submitted and confirmed to be positive by dFA. Suspected cases were defined as those in which samples were collected from animals exhibiting a change of behavior with a history of contact with a confirmed case and whose brain tissue was submitted to the laboratory but had not been tested by dFA. The available data were entered, cleaned, validated, and descriptively analyzed in a Microsoft Excel spreadsheet (Microsoft Office Excel, 2019). The positivity rate was calculated as the percentage of the samples confirmed positive by dFA out of the total submitted for confirmatory diagnosis. Thus, the annual rabies positivity rate was calculated using the formula C/S × 100, where C = confirmed and S = submitted.

### Brain tissue sample collection

**Fig. 1:**
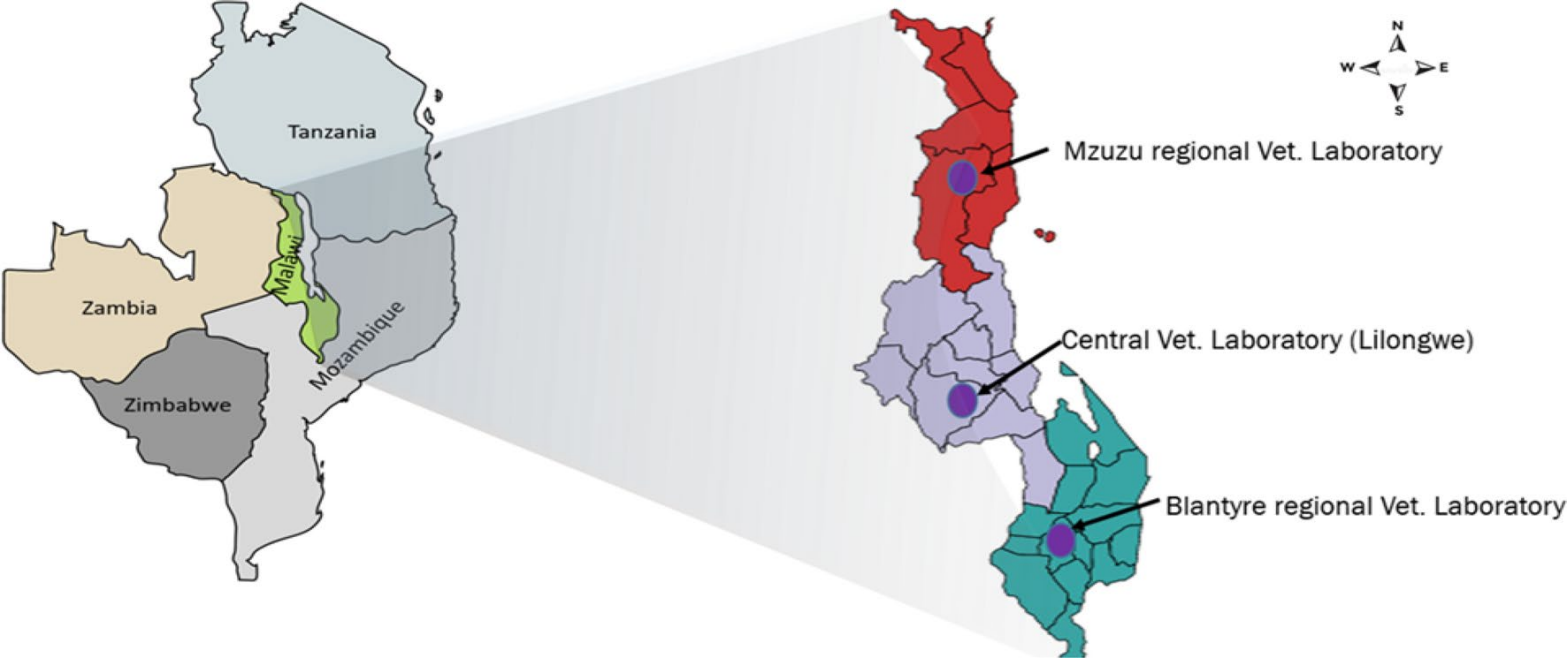
Map of Malawi showing the geographical locations of the veterinary laboratories and the regions from which dFA-positive samples originated. The purple, grey, and red colored regions correspond to the Northern, Central, and Southern region of Malawi, respectively.

The study used archived viable samples available at Malawi’s CVL and did not involve active, systematic sampling. Rabies-suspected samples were submitted from district agricultural administrative areas through regional veterinary laboratories for confirmatory diagnosis. Although the samples were not collected evenly throughout the country, the findings of this study can nevertheless provide baseline information for further studies on rabies in Malawi. Between 2019 and 2021, a total of 46 viable rabies-suspected brain samples from cats (1), cattle (5), and dogs (40) from the three regions of Malawi were collected (Supplementary Table S1). The samples were submitted to the Mzuzu (Northern region), Blantyre (Southern region), and Central (Central region) veterinary laboratories from 14 districts for the purpose of rabies diagnosis (Fig. 1). Thereafter, the samples were transported to the School of Veterinary Medicine, University of Zambia (UNZA), for molecular characterization.

### RNA extraction and nested PCR

Total RNA was extracted from RABV-positive brain samples using a QIAamp Viral RNA Mini Kit (QIAGEN, Hilden, Germany) following the manufacturer’s instructions. A nested PCR assay was performed using the outer primers RN1 (5-CTACAATGGATGCCGAC-3) and RN2 (5-GAGTCACTCGAATATTGC-3) and the inner primers RN3 (5-GACATGTCCGGAAGACTGG-3) and RN4 (5-GTATTGCCTCTCTAGCGGTG-3) [45] for the N gene and RAVGF (5-CAAGGAAAGATGGTTCCTCAG-3) and the outer primers RAVGR (5-TCACAGTCTGGTCTCACCTCCAC-3) [13] and the inner primers GF (5-CCATCATGACCACCAAGTC-3) and GR (5-TTACAGCTTGGTCTCACC-3) [14] for the G gene. The primers RN1, RN2, RN3, and RN4 correspond to N gene positions 66–82, 1402–1419, 319–337, and 823–842, respectively, and the primers RAVF, RAVG, GF, and GR correspond to G gene positions 3309–3329, 4870–4892, 923–941, and 1558–1575, respectively, of the Pasteur virus genome sequence (NC 001542.1). In the primary PCR, the target gene fragments were amplified using a Superscript One-Step RT-PCR Kit (Invitrogen, California, USA), employing outer primers and thermal cycling conditions reported previously [45]. Nested PCR was performed using an Extaq HS PCR Kit (Takara Bio Inc, Shinga, Japan) with the appropriate inner primer sets. PCR products of approximately 500 bp and 650 bp for the N and G gene, respectively, were electrophoresed in a 1.5% agarose gel stained with ethidium bromide and visualized under UV light.

### PCR product purification and direct sequencing

The nested PCR products were purified using a Monofas Purification Kit (GL Sciences, Kyoto, Japan) as per the manufacturer's instructions. Direct sequencing was done using a Big Dye Terminator v3.1 system (Applied Biosystems, California, USA) and the inner primer sets for the N and G genes [14, 45]. The direct sequencing products were purified by the ethanol precipitation method and sequenced on an ABI 3500 Genetic Analyzer (Applied Biosystems, California, USA).

### Sequence analysis

All of the sequences obtained were subjected to BLAST analysis on the NCBI website (https://blast.ncbi.nlm.nih.gov/Blast.cgi) and then assembled and edited using the ATGC application incorporated in Genetyx Ver. 12 (GENETYX Corporation, Tokyo, Japan). The final sequence length after trimming and editing was 460 bp for the N gene and 609 bp for the G gene. In addition, RABV sequences from various parts of the world representing different lineages and phylogroups were downloaded from NCBI GenBank database for the purpose of identifying the lineages and examining the diversity of RABV sequences from different regions and hosts in Malawi. Thereafter, RABV sequences from Malawi and reference sequences from GenBank were aligned using ClustalW1.6, and the resulting multiple sequence alignment fasta file was converted to mega file format using MEGA ver. 6 [46]. Model selection and construction of maximum-likelihood (ML) phylogenetic trees based on the Tamura 3-parameter model [47] and 1,000 bootstrap replicates [48] were performed using MEGA ver. 6 [46]. Evolutionary rate differences among sites were determined using a discrete gamma distribution with five categories. In addition, DNA polymorphisms (nucleotide diversity) were calculated using DnaSP ver. 6 [49].

## Results

### Cases, vaccinations and diagnostic reports

**Fig. 2:**
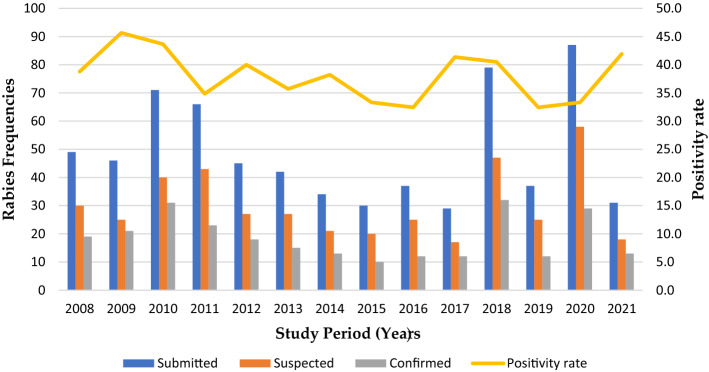
Number of submitted reports, suspected cases, and confirmed cases, positivity rate of rabies cases in Malawi from 2008 to 2021

A total of 683 rabies reports were obtained from across the country over a period of 14 years (2008-2021). The analysis showed that report submission was highest in 2010, and thereafter, there was a decline from 2012 to 2017. The year 2017 had the lowest number of submissions (*n* = 29) (Table 1, Fig. 2). The trend then started to increase again, with 79 and 87 submissions in 2018 and 2020, respectively. However, there was a decline in the number of submissions in 2019 and 2021. The largest number of cases were in domestic dogs (*n* = 435), followed by cattle (*n* = 94), cats (*Felis catus*) (*n* = 38), goats (*Capra hircus*) (*n* = 27), hyenas (*Crocuta crocuta*) (*n* = 26), and side-striped jackals (*n* = 17) (Table 1). Within each year, a number of submissions were confirmed as either positive or negative for rabies (Fig. 2). The positivity rate remained relatively constant throughout the study period and was the highest in 2009 and the lowest in 2019. Overall, the rabies reports indicate that domestic dogs are the main source of infection of other animals, especially livestock. Although suspected rabies cases were submitted to the CVL for diagnosis, reporting and diagnosis were inconsistent (Table 1, Fig. 2).

**Table 1:** Reports of suspected rabies cases in different animal species obtained by the Central Veterinary Laboratory for Rabies Diagnosis in Malawi from 2008 to 2021

Species	Rabies report submissions for each species per year														
	2008	2009	2010	2011	2012	2013	2014	2015	2016	2017	2018	2019	2020	2021	Total
Dog	23	22	31	47	28	24	22	23	25	16	56	26	69	23	435
Cat	5	5	4	2	5	2	1	1	3	2	3	5	0	0	38
Cattle	6	16	8	11	6	7	6	3	3	9	6	3	4	6	94
Monkey	3	2	0	0	0	1	0	0	1	0	0	0	1	0	8
Hyena	2	1	8	0	4	0	0	1	1	0	3	1	5	0	26
Rabbit	1	0	0	0	0	0	0	1	0	0	0	0	0	0	2
Goat	6	0	7	5	0	0	0	0	0	0	4	0	5	0	27
Jackal	2	0	4	0	0	2	2	0	2	0	2	0	1	2	17
Wild pig	1	0	7	0	0	0	0	1	1	0	0	0	0	0	10
Human	0	0	0	0	0	0	0	0	0	2	0	2	2	0	6
Sheep	0	0	2	0	0	0	0	0	0	0	0	0	0	0	2
Honey badger	0	0	0	1	0	2	0	0	0	0	0	0	0	0	3
Bat	0	0	0	0	2	0	0	0	0	0	0	0	0	0	2
Zebra	0	0	0	0	0	2	0	0	0	0	3	0	0	0	5
Donkey	0	0	0	0	0	2	0	0	0	0	2	0	0	0	4
Mongoose	0	0	0	0	0	0	3	0	0	0	0	0	0	0	3
Rat	0	0	0	0	0	0	0	0	1	0	0	0	0	0	1
Total	49	46	71	66	45	42	34	30	37	29	79	37	87	31	683

Anti-rabies vaccination reports from DAHLD showed that dogs and cats were the major recipients of rabies vaccines (Table 2). No other animals were reported to have been vaccinated during the study period. The reports further showed a steady increase in vaccine procurement from 2008 to 2012 and 2014 and 2015 (Table 2). DAHLD procured the listed doses of vaccine centrally, and these were distributed proportionally to districts. However, there were many other nongovernmental organizations (NGOs) and donors who supplied additional doses to some districts that were not recorded at DAHLD. The highest number of doses was procured in 2017 and the lowest in 2016 (Table 2). The coverage of rabies vaccination varied widely across the study period, with the highest vaccination coverage (60%) in 2008 and 2013 and the lowest coverage (5%) in 2016 (Table 2). Overall, vaccination coverage was inconsistent despite the fact that the number of vaccine doses procured per year was increasing.

**Table 2:** Dosages of anti-rabies vaccine procured and vaccination coverage in Malawi from 2008 to 2021

Year	Population of dogs and cats	Vaccine doses procured	% Procured	Vaccinated dogs and cats	% Vaccinated
2008	530,072	100,000	19	316,590	60
2009	695,487	120,000	17	362,115	52
2010	589,104	120,000	20	235,000	40
2011	1,221,414	150,000	12	529,240	43
2012	767,672	200,000	26	171,289	22
2013	498,174	170,000	34	297,000	60
2014	888,986	225,000	25	257,894	29
2015	917,941	290,000	32	267,351	29
2016	881,582	45,000	5	44,360	5
2017	783,885	350,000	45	119,816	15
2018	508,934	225,000	44	198,234	39
2019	569,862	300,000	53	209,775	37
2020	571,090	240,000	42	229,067	40
2021	575,379	200,000	35	187,593	33

The overall rabies positivity rate based on dFA results was 38.1% (260/683) (CI: 34.43-41.84). The highest positivity rate of 50% (3/6) was in humans, followed by livestock, with 40.3% (52/129), and pets, with 37.8% (179/473) (Table 3). The positivity rate in cattle and dogs was 39.4% and 39%, respectively (Table 3). In contrast, dogs contributed a higher proportion of the overall positivity (65.4%; 170/260; CI: 59.22-71.09) than cattle (20%; 52/260; CI: 15.42-25.49). Sheep (*Ovis aries*) and donkeys (*Equus asinus*) had positivity rates of 50%. Among the wildlife, side-striped jackal and hyena had higher positivity rates of 47.1% (8/17) and 34.6% (9/26), respectively. Cases in other domestic animals and wildlife species made up the minority of the reported or confirmed cases (Table 3). Overall, the case reports demonstrated that domestic dogs are the main infected host likely to be a source of RABV infection of domestic animals and humans. With regard to wildlife, both side-striped jackals and hyenas had higher positivity rates (Table 3).

**Table 3:** Distribution of reported and confirmed cases of rabies in Malawi across host categories and species from 2008 to 2021

Host category	Host	Number reported	Number confirmed	Positivity (%)	95% CI
Pets		473	179	37.8	33.48-42.03
Livestock		129	52	40.3	31.88-49.33
Wildlife		75	26	34.7	24.29-46.62
Humans		6	3	50.0	18.76-81.24
Total		683	260	38.1	34.43-41.84
Pets	Dogs	435	170	39.1	34.49-43-86
	Cats	38	9	23.7	12.03-40.61
Livestock	Cattle	94	37	39.4	34.49-43.86
	Goats	27	12	44.4	26.04-64.36
	Sheep	2	1	50	9.45-90.55
	Donkeys	4	2	50	15.00-84.99
	Rabbits	1	0	0	0.00-94.54
Wildlife	Hyenas	26	9	34.6	17.94-55.64
	Side-striped jackals	17	8	47.1	23.86-71.47
	Monkeys	8	1	12.5	0.66-53.32
	Wild pigs	10	3	30	8.09-64.63
	Zebras	5	2	40	7.26-82.96
	Honey badgers	3	1	33.3	1.76-87.67
	Mongooses	3	1	33.3	1.76-87.67
	Bats	2	1	50	9.45-90.55
	Rats	1	0	0	0.00-94.54

CI = confidence interval

### Nested RT-PCR and direct sequencing

Out of a total of 46 RNA samples (dog = 40, cow = 5, and cat = 1) extracted from rabies-suspected brain tissues, only 23 were positive for both genes. An additional 12 and six samples were positive for the N and G gene, respectively, and six samples that were positive for the N gene were negative for the G gene (Supplementary Table S1). In total, 35 (dog = 31, cow = 3, cat =1) and 29 (dog = 24, cow = 4, cat = 1) samples were positive by nested RT-PCR for the RABV N and G gene, respectively, and the sequences of these genes have been deposited in the DNA Data Base of Japan (DDBJ) with serial accession numbers LC682823 to LC682857 (N gene) and LC683159 to LC683187 (G gene) (Supplementary Table S1). Among the positive samples, the number of owned, stray, vaccinated, and unvaccinated animals was 23, 12, eight, and 27, respectively (Supplementary Table S1). The number of the male and female dogs was 20 and 15 respectively, and the number of young and adult dogs was eight and 27, respectively (Supplementary Table S1). Overall, out of the 46 samples tested by RT-PCR, there was a higher proportion of unvaccinated animals than vaccinated animals, and the majority of these were males.

### Phylogenetic analysis

**Fig. 3:**
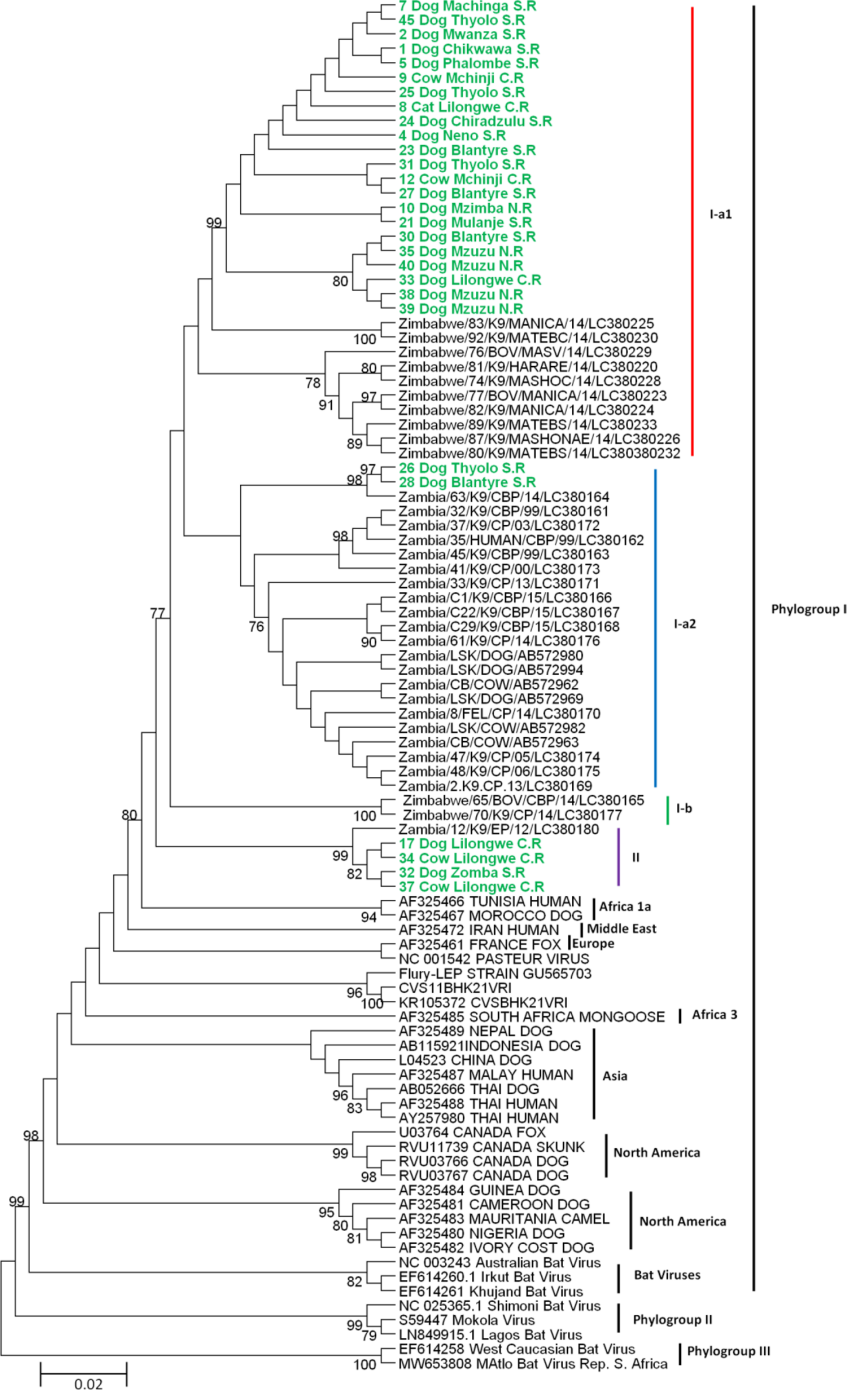
Phylogenetic analysis based on a 609-nt fragment of the glycoprotein (G) gene of RABV isolates from Malawi, constructed using MEGA ver. 6 with 1000 bootstrap replicates. The maximum-likelihood tree was generated using the Tamura 3-parameter model with gamma distribution rates across sites. Sequences from Malawi are shown in green and bold. "NR", "CR", and "SR" at the end of the sequences from this study denote the Northern region, Central region, and Southern region, respectively.

Phylogenetic analysis based on the nucleotide sequences of a portion of the G gene revealed two clusters, namely, I and II, belonging to the Africa 1b lineage in phylogroup I (Fig. 3). While cluster II occurred as a single cluster, cluster I was further divided into subclusters I-a and I-b, with subcluster 1-a being further divided into minor clusters 1-a1 and I-a2 (Fig. 3). In subcluster I-a1, sequences from Malawi (n = 22) clustered together in close proximity to reference sequences from Zimbabwe (Fig. 3), while subcluster I-a2 consisted mainly of sequences from Zambia and only two sequences (28 Dog Blantyre S.R and 26 Dog Thyolo S.R) from the southern region of Malawi (Fig. 3). On the other hand, cluster II mainly consisted of sequences (n = 4) from Malawi, with one sequence (Zambia/12/K9/EP/12/LC380180) from the eastern province of Zambia. Overall, the sequences from this study clustered according to their geographical origin, except for the sequences 28 Dog Blantyre S.R and 26 Dog Thyolo S.R from Malawi, which clustered with Zambian sequences in subcluster I-a2, and sequence Zambia/12/K9/EP/12/LC380180 from Zambia, which clustered with sequences from the Lilongwe and Zomba regions of Malawi in cluster II (Fig. 3). A DNA polymorphism rate of 0.13661 (13.66%) was observed among the sequences.

**Fig. 4:**
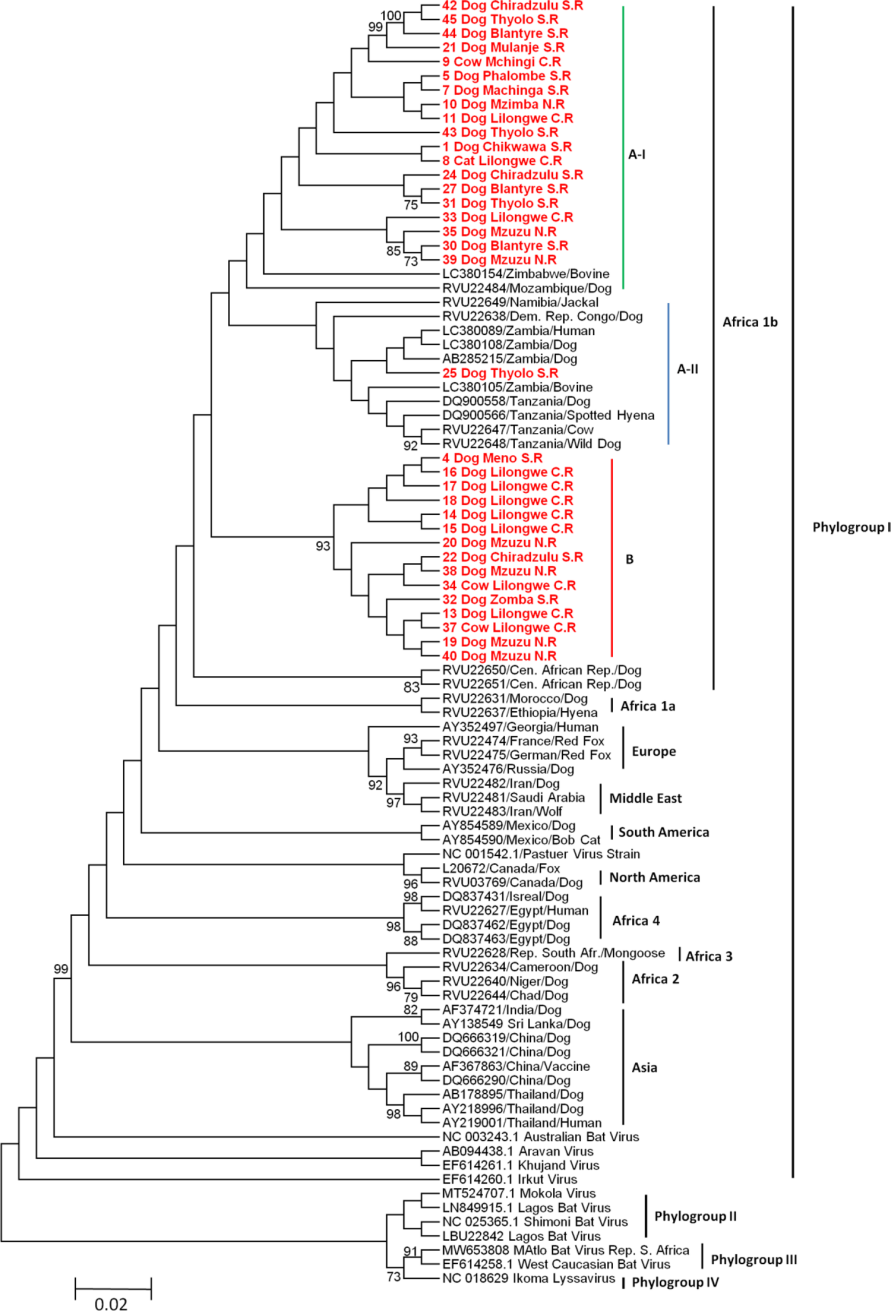
Maximum-likelihood phylogenetic tree constructed based on partial nucleoprotein (N) gene nucleotide sequences (460 nt) of RABV isolates from the republic of Malawi and other areas of the world. The sequences from different regions of Malawi are shown in bold and red, while the remaining reference sequences are in black. The phylogenetic tree was constructed using MEGA ver. 6 with the Tamura 3-parameter model with 5 gamma distribution rates among sites and 1000 bootstrap replicates. NR, CR, and SR in the Malawi sequence names correspond to the Northern region, Central region, and Southern region, respectively.

Similarly, phylogenetic analysis based on N gene sequences from different regions of Malawi showed that these isolates clustered in phylogroup I, specifically with the Africa 1b reference sequences (Fig. 4). Within the Africa 1b cluster, sequences from Malawi were found in clusters A and B. Cluster A was further divided into minor clusters A-I, comprising Malawian sequences (n = 19) and two reference sequences from Zimbabwe (LC380154) and Mozambique (RVU22484), while minor cluster A-II consisted mainly of reference sequences from the Central African Republic, Democratic Republic of Congo, Mozambique, Namibia, Tanzania, Zambia, and Zimbabwe, together with one sequence (25 Dog Thyolo S.R) from the Thyolo district of Malawi (Fig. 4). On the other hand, cluster B consisted exclusively of sequences (n = 15) from this study. Phylogenetic analysis based on the N gene showed that two strains of RABV belonging to the Africa 1b lineage might be circulating in different regions and hosts in Malawi. A DNA polymorphism rate of 0.13764 (13.76%) was observed among the sequences.

## Discussion

Rabies endangers the lives of about half of the world population, and WHO, the World Organization for Animal Health (WOAH), and the Food and Agriculture Organization (FAO) have therefore decided to set the goal of eliminating dog-mediated human rabies deaths by 2030 [50, 51]. In pursuit of this goal, this study was conducted to examine the current status of reporting and submission of brain samples for diagnosis and determination of the positivity rate and the molecular epidemiology of rabies in different host species and regions of Malawi. This information is pertinent for directing national efforts towards the eradication of dog-mediated human rabies.

Since the study in 1995 by Edelsten [52] on the growing positivity rate of rabies, the disease has caused death in humans and animals, with most cases remaining undiagnosed or misdiagnosed [37, 38, 41]. Despite underreporting of human rabies cases, several human deaths due to dog-mediated rabies have been reported in the southern region of Malawi [37, 38, 41], and humans continue to suffer, as demonstrated by the reported human rabies cases (Table 3). The confirmed cases in wildlife and livestock have increased the risk of rabies in humans, thus drawing special attention from the government of Malawi. In order to reverse this trend, the DAHLD in Malawi approved a policy that recognizes rabies as an endemic disease [53]. Malawi has also embarked on annual free mass anti-rabies vaccination and a campaign of neutering of dogs and cats in order to reduce the burden of rabies and control the pet population in the community. Furthermore, the DAHLD recommends that at least 80% of the dog and cat population should be vaccinated each year and that vaccination should be provided for free during rabies campaign periods [50, 53]. Unfortunately, this coverage was not achieved during the study period, which possibly led to the increase in the number of suspected and confirmed cases as well as the prevalence of rabies, with dogs accounting for the highest number of cases (Table 3) [52]. It is also important to note that in 2017, even though a total of 350,000 doses were procured, vaccination coverage of only 15% was achieved. This could be due to poor sensitization or implementation. Furthermore, the number of doses procured each year did not correlate with the target population (Table 2). This is a typical expression of underreporting and inconsistencies in report submission from the districts, because rabies vaccine doses are procured centrally for subsequent distribution to the districts, in amounts based on the current trends in rabies cases. Thus, if some information is missing, the wrong number of vaccine doses will be procured. The difficulties in reporting may be explained further by a lack of information about vaccinations conducted outside the designated rabies vaccination period and private rabies campaigns that might not be included in the national reports.

The current situation of dog vaccination, in as much as it is free, is inadequate and lags behind the 80% target. Thus, a large proportion of dogs and cats are left unprotected and vulnerable to devastating RABV infections. Regrettably, the inadequate coverage has been masked by low rates of reporting and submission of brain samples for rabies diagnosis (Table 1). Furthermore, the failure to diagnose some of the brain samples that were submitted could be due to a lack of reagents or the samples arriving at the laboratory in a decomposed state, rendering them unusable. This signifies that the passive surveillance approach currently in use has significant limitations in achieving rabies elimination, since reporting is based only on active clinical signs, with or without laboratory-based confirmation, as evidenced by the large number of suspected cases compared to the number of confirmed cases (Fig. 2). This further implies that although diagnosis is carried out, it is done inconsistently, possibly due to inadequate resources, thus hindering the effective diagnosis of rabies and negatively affecting the reporting processes. Interestingly, there were a larger number of reports in 2020 than in other years, and this could be a positive indication of improved community awareness and reporting mechanisms being implemented in the country [40]. The reporting of rabies in wildlife was also inconsistent, as evidenced by the fact that cases were reported in certain wildlife species in some years, but not in other years (Table 1). Failure of periodic and consistent reporting of rabies cases from all possible pockets of RABV reservoirs signifies that the current strategies for mitigating rabies in Malawi are not efficient enough to eliminate rabies by 2030. Therefore, future government efforts should consider a combination of passive and active surveillance to strengthen rabies monitoring and reporting in Malawi.

The rabies positivity rate in this study was higher in dogs than in other species. This is in agreement with previous studies [4, 8] and suggests that rabies is mainly transmitted by dogs, with spillover infections in livestock and humans. With regard to wildlife, side-striped jackals and hyenas accounted for 6.5% of the wildlife rabies cases, in contrast to the previously reported 5% [52]. In addition, this study also shows, for the first time, the circulation of RABV in bats (*Miniopterus natalensis*), honey badgers (*Mellivora capensis*), wild pigs (*Sus scrofa*), monkeys (*Cercopithecus pygerythrus*), mongooses (*Helogale parvula*), and zebras (*Equus burchellii*) in Malawi (Table 3) and further suggests spillover of RABV from dogs and side-striped jackals to domestic animals and wildlife. The involvement of bats in the circulation of RABV in wildlife indicated the need for active surveillance of migrating bats. It has been reported that all lyssaviruses, including RABV, are likely to have originated in bats [32]. With the exception of Mokola lyssavirus (MOKV), all lyssaviruses have been isolated from migrating bats [32, 54]. Thus, periodic studies of migrating bats are essential for understanding both RABV and chiropteran host ecology and for evaluating the possible spillover transmission to dog and human populations. This, therefore, warrants the consideration of additional monitoring and vaccination strategies in certain wildlife species for effective prevention and control of rabies.

In order to strengthen the data obtained from case reports and improve the resolution of the current trends of rabies in Malawi, RNA was extracted from rabies-suspected brain tissues and screened for the presence of the RABV genome using RT-PCR followed by phylogenetic analysis of partial sequences of the G and N genes (Fig. 3 and Fig. 4). The failure to detect the whole RABV genome in samples that were positive for one gene and not on the other, i.e., the six samples that tested negative for the G gene but positive for the N gene, could be attributed to poor quality of the RNA or improper storage of samples, as reported previously [16, 55]. A high level of nucleotide sequence diversity was also observed in the regions of the N and G genes analyzed. Phylogenetic analysis of the G gene revealed the presence of two strains that are circulating in different hosts and geographical locations in Malawi (Fig. 3). These strains are not entirely restricted to specific geographical regions of Malawi but instead are distributed in a way similar to strains in Zambia [16] and Zimbabwe [17]. In addition, the RABV sequences from Malawi and other regions clustered according to their country of origin, with the exception of the sequences 28 Dog Blantyre S.R and 26 Dog Thyolo S.R, which clustered with sequences from Zambia, and sequence Zambia/12/K9/EP/12/LC380180 from Zambia, which clustered with sequences from the Lilongwe and Zomba areas of Malawi (Fig. 3). These viruses could have been introduced into Malawi or Zambia via transboundary migration of reservoir animals [56, 58] and/or cross-border trade of animals. Both scenarios are plausible, as Malawi and Zambia share a border that is not restricted by physical barriers or strict enforcement of border control. Furthermore, the similarities of the cultures of these two countries allows for easier movement of people and their animals from one country to the other. Phylogenetic analysis based on the N gene indicated that the RABV strains circulating in different hosts and regions of Malawi belong to the Africa 1b lineage (Fig. 4), which is prevalent in eastern and southern Africa [16, 17, 54, 56, 57] and further demonstrated the presence of at least two strains that are possibly equally distributed within the country (Fig. 4). The detection of members of cluster B (Fig. 4), a separate genetic lineage, suggests the circulation of a discrete strain of RABV in all three regions of Malawi. Unvaccinated domestic dogs could account for the discrete circulation of RABV in Malawi [5]. In this study, RABV sequences clustered together according to geography, irrespective of host origin, providing evidence that similar RABV strains circulate locally in certain ecological landscapes that, in some regions, extend across national borders, and this is compatible with cross-species transmission. Since the strains isolated from livestock are clearly similar to those in dogs and humans, dogs are the most likely source of infection in all hosts and regions of Malawi (Fig. 3). Dogs live freely with humans and livestock, particularly in rural areas, and, unfortunately, most of them are unvaccinated. These interactions, coupled with human population growth and ineffective control of stray dogs and rabies, has resulted in an increase in dog bite cases, leading to higher rabies positivity rate in humans, livestock, and wildlife [37, 38], as evidenced by the rabies reports submitted (Tables 2 and 3). Furthermore, it is possible that other strains of RABV are circulating in other hosts in Malawi that were not examined in this study. Thus, more-robust molecular epidemiological studies focusing on whole genome sequences are warranted.

## Conclusions

This study provides data on the positivity rate and molecular epidemiology of RABV in different host species in all regions of Malawi and shows that low and inconsistent vaccination coverage is exacerbated by underreporting and insufficient documentation of rabies cases. The data suggest that the strains of RABV currently circulating in different geographical areas are transmitted by unvaccinated dogs. Thus, it is recommended that increased efforts be made to vaccinate dogs and cats, with special consideration of the frequency and coverage of vaccination campaigns. Overall, the observations in this study call for increased periodic surveillance of migrating bats, monitoring of cross-border movement, dog/pet vaccination, reporting and timely submission of samples from suspected rabies cases for laboratory analysis in order to eliminate dog-mediated rabies in humans in Malawi by 2030.

## Supplementary Information

Below is the link to the electronic supplementary material.

Supplementary file1 (DOCX 34 KB)

## Data Availability

All data have been provided in the article and as supplementary materials.
