# Supplementary material for: Current status and molecular epidemiology of rabies virus from different hosts and regions in Malawi
Source: Arch Virol. 2023 Jan 12;168(2):61. doi: 10.1007/s00705-022-05635-z (PMC9834359; doi:10.1007/s00705-022-05635-z)
Supplement: Supplementary file 1 — Supplementary file1 (DOCX 34 KB) [file 705_2022_5635_MOESM1_ESM.docx]

Supplementary Table S1. Suspected rabies samples collected from different regions of Malawi from 2019 - 2021 used for molecular analysis in this study

| Sample ID | Date of collection | Status | Species | Age | Sex | Vaccination status | District | Region | Accession | Accession |
| --- | --- | --- | --- | --- | --- | --- | --- | --- | --- | --- |
| 1DogChikwawaSR | 16/01/2019 | Owned | Canine | Adult | Male | No | Chikwawa | Southern | LC682823 | LC683159 |
| 2DogMwanzaSR | 26/06/2019 | Owned | Canine | Adult | Male | No | Mwanza | Southern |  | LC683160 |
| 4DogMenoSR | 13/01/2020 | Stray | Canine | Adult | Male | No | Meno | Southern | LC682824 | LC683161 |
| 5DogPhalombeSR | 22/02/2020 | Stray | Canine | Young | Male | No | Phalombe | Southern | LC682825 | LC683162 |
| 6DogZombaSR | 16/4/2020 | Stray | Canine | Adult | Female | No | Zomba | Southern |  |  |
| 7DogMachingaSR | 29/07/2020 | Stray | Canine | Adult | Female | No | Machinga | Southern | LC682826 | LC683163 |
| 8CatLilongweCR | 15/01/2019 | Owned | Feline | Adult | Male | Yes | Lilongwe | Central | LC682827 | LC683164 |
| 9CowMchinjiCR | 15/01/2019 | Owned | Bovine | Adult | Female | No | Mchinji | Central | LC682828 | LC683165 |
| 10DogMzimbaNR | 18/03/2019 | Stray | Canine | Adult | Female | No | Mzimba | Northern | LC682829 | LC683166 |
| 11DogLilongweCR | 28/03/2019 | Owned | Canine | Adult | Male | No | Lilongwe | Central | LC682830 |  |
| 12CowMchinjiCR | 14/06/2019 | Owned | Bovine | Adult | Female | No | Mchinji | Central |  | LC683167 |
| 13DogLilongweCR | 26/06/2019 | Owned | Canine | Adult | Male | Yes | Lilongwe | Central | LC682831 |  |
| 14DogLilongweCR | 28/06/2019 | Owned | Canine | Adult | Male | No | Lilongwe | Central | LC682832 |  |
| 15DogLilongweCR | 28/06/2019 | Owned | Canine | Adult | Female | Yes | Lilongwe | Central | LC682833 |  |
| 16DogLilongweCR | 28/06/2019 | Owned | Canine | Adult | Male | Yes | Lilongwe | Central | LC682834 |  |
| 17DogLilongweCR | 14/7/2019 | Owned | Canine | Adult | Male | No | Lilongwe | Central | LC682835 | LC683168 |
| 18DogLilongweCR | 13/08/2019 | Stray | Canine | Adult | Female | No | Lilongwe | Central | LC682836 |  |
| 19DogMzuzuNR | 17/10/2019 | Stray | Canine | Adult | Male | No | Mzuzu | Northern | LC682837 |  |
| 20DogMzuzuNR | 17/10/2019 | Owned | Canine | Adult | Male | No | Mzuzu | Northern | LC682838 |  |
| 21DogMulanjeSR | 15/09/2020 | Stray | Canine | Young | Female | No | Mulanje | Southern | LC682839 | LC683169 |
| 22DogChiradzuluSR | 8/7/2021 | Owned | Canine | Adult | Female | Yes | Chiradzulu | Southern | LC682840 |  |
| 23DogBlantyreSR | 12/7/2021 | Owned | Canine | Adult | Female | Yes | Blantyre | Southern |  | LC683170 |
| 24DogChiradzuluSR | 13/07/2021 | Owned | Canine | Adult | Male | Yes | Chiradzulu | Southern | LC682841 | LC683171 |
| 25DogThyoloSR | 14/07/2021 | Stray | Canine | Young | Male | No | Thyolo | Southern | LC682842 | LC683172 |
| 26DogThyoloSR | 15/07/2021 | Stray | Canine | Adult | Male | No | Thyolo | Southern |  | LC683173 |
| 27DogBlantyreSR | 16/07/2021 | Stray | Canine | Young | Male | No | Blantyre | Southern | LC682843 | LC683174 |
| 28DogBlantyreSR | 16/07/2021 | Stray | Canine | Young | Male | No | Blantyre | Southern |  | LC683175 |
| 29DogThyoloSR | 17/07/2021 | Stray | Canine | Adult | Female | No | Thyolo | Southern |  |  |
| 30DogBlantyreSR | 21/07/2021 | Stray | Canine | Adult | Female | No | Blantyre | Southern | LC682844 | LC683176 |
| 31DogThyoloSR | 21/07/2021 | Stray | Canine | Adult | Female | No | Thyolo | Southern | LC682845 | LC683177 |
| 32DogZombaSR | 26/07/2021 | Stray | Canine | Adult | Male | No | Zomba | Southern | LC682846 | LC683178 |
| 33DogLilongweCR | 19/07/2021 | Owned | Canine | Young | Male | No | Lilongwe | Central | LC682847 | LC683179 |
| 34CowLilongweCR | 30/06/2021 | Owned | Bovine | Adult | Male | No | Lilongwe | Central | LC682848 | LC683180 |
| 35DogMzuzuNR | 6/6/2021 | Owned | Canine | Adult | Female | Yes | Mzuzu | Northern | LC682849 | LC683181 |
| 36CowLilongweCR | 11/7/2021 | Owned | Bovine | Adult | Female | No | Lilongwe | Central |  |  |
| 37CowLilongweCR | 8/6/2021 | Owned | Bovine | Adult | Female | No | Lilongwe | Central | LC682850 | LC683182 |
| 38DogMzuzuNR | 5/5/2021 | Owned | Canine | Adult | Male | No | Mzuzu | Northern | LC682851 | LC683183 |
| 39DogMzuzuNR | 8/5/2021 | Owned | Canine | Adult | Female | No | Mzuzu | Northern | LC682852 | LC683184 |
| 40DogMzuzuNR | 8/5/2021 | Owned | Canine | Adult | Male | No | Mzuzu | Northern | LC682853 | LC683185 |
| 41DogBlantyreSR | 28/07/2021 | Stray | Canine | Young | Male | No | Blantyre | Southern |  | LC683186 |
| 42DogChiradzuluSR | 5/8/2021 | Own | Canine | Adult | Female | Yes | Chiradzulu | Southern | LC682854 |  |
| 43DogThyoloSR | 10/8/2021 | Own | Canine | Young | Female | No | Thyolo | Southern | LC682855 |  |
| 44DogBlantyreSR | 10/8/2021 | Own | Canine | Young | Male | No | Blantyre | Southern | LC682856 |  |
| 45DogThyoloSR | 12/8/2021 | Own | Canine | Young | Female | No | Thyolo | Southern | LC682857 | LC683187 |
| 46DogChiradzuluSR | 14/08/2021 | Own | Canine | Adult | Male | No | Chiradzulu | Southern |  |  |
| 47DogThyoloSR | 18/08/2021 | Own | Canine | Young | Female | Yes | Thyolo | Southern |  |  |
